# Surgical field visualization and feasibility of accessory lung lobectomy using a pretied ligature loop via single incision thoracoscopy in canine cadavers

**DOI:** 10.3389/fvets.2025.1682911

**Published:** 2025-11-25

**Authors:** Chaewon Kim, Sangjun Lee, Suyoung Heo

**Affiliations:** Department of Veterinary Surgery, College of Veterinary Medicine, Jeonbuk National University, Iksan, Republic of Korea

**Keywords:** thoracoscopy, subxiphoid approach, CO_2_ insufflation, accessory lung lobectomy, pretied ligature, small dog

## Abstract

**Introduction:**

Thoracoscopic surgery is a widely accepted minimally invasive technique in veterinary medicine. However, its application in small dogs is limited by the narrow thoracic space, difficulty accessing midline structures, and the requirement for one-lung ventilation. To overcome these limitations, a single-incision subxiphoid approach with low-pressure CO_2_ insufflation may offer improved visualization and access, but objective, quantitative evidence supporting its feasibility remains insufficient.

**Materials and methods:**

Eight canine cadavers (6–10 kg) underwent subxiphoid single-port thoracoscopy under mechanical ventilation with non-insufflation and 3 mmHg CO_2_ insufflation. Visualization quality was quantitatively evaluated using image analysis, and lung lobe accessibility was scored. The feasibility and safety of accessory lung lobectomy using a pretied ligature loop was assessed, including air leakage testing.

**Results:**

CO_2_ insufflation at 3 mmHg group significantly enhanced thoracoscopic visualization compared to non-insufflation group, particularly by reducing visual obstruction from the left middle and right caudal lung lobes. All lung lobes were accessible through a single incision, though the accessory lung lobe was more difficult to approach (mean exploration score: 0.59). Accessory lung lobectomy using a pretied loop was successfully completed in all cadavers without evidence of air leakage, with an average procedure time of 921 s.

**Discussion:**

A single-incision subxiphoid thoracoscopic approach combined with low-pressure CO_2_ insufflation significantly improves visualization and instrument maneuverability in small-breed canine cadavers. This method also enables complete lung lobe exploration and allows safe and effective accessory lung lobectomy using a pretied ligature loop.

## 1 Introduction

Minimally invasive techniques, such as laparoscopy and thoracoscopy, have advanced rapidly in both human and veterinary medicine and are widely used to manage pulmonary and mediastinal disorders in dogs (1–5). However, thoracoscopy in small dogs and cats remains challenging due to limited intrathoracic workspace. Surgical access to midline structures, including the cranial mediastinum and accessory lung lobe, is particularly demanding because of their anatomical position and proximity to the heart and great vessels (6–9). One-lung ventilation (OLV), the standard method for improving visualization by collapsing the lungs, is technically demanding in these patients due to anatomical constraints (3, 5, 10). Low-pressure carbon dioxide (CO₂) insufflation at 3 mm Hg has been proposed as a simpler alternative to OLV, and previous studies have also evaluated the combined use of OLV and CO₂ insufflation to further improve visualization and reduce lung motion during thoracoscopy (11). Recent evidence has quantitatively demonstrated that this combination increases the intrathoracic working space and enhances operative visibility in dogs (12). Optimal access route selection is critical to mitigate spatial restrictions. Subxiphoid thoracoscopy with the animal in dorsal recumbency has been suggested to enhance access to the central thoracic structures compared with the traditional lateral intercostal approach (13, 14). A single incision at this site may reduce intercostal nerve damage by eliminating additional ports while providing access to both pleural cavities (13–15). Despite these theoretical advantages, quantitative data on operative speed, image quality, and the ability to survey the entire pulmonary surface using this technique in dogs are scarce. The efficiency of performing complex interventions, such as lung-lobe resection, through a single subxiphoid portal remains uncertain and depends significantly on instrument selection (14). Commercial endoscopic staplers (e.g., Endo GIA), commonly used for thoracoscopic lobectomy, are often impractical for toy-breed dogs because of spatial constraints (16). In addition, their high cost can be a limitation. As a cost-effective alternative, occlusion of pulmonary vessels and bronchi using pretied loop ligatures has been proposed, though clinical validation of this approach remains limited in small animals, including dogs and cats (17–19). A recent study also indicated that restricting the operative setup to two or three long, slender instruments reduces crowding and improves maneuverability—an important advantage when applying loop ligatures in confined regions such as the accessory lung lobe (14). Given these knowledge gaps, the present study was designed to evaluate the feasibility of single-incision subxiphoid thoracoscopy in small dogs. The specific objectives were to (1) quantitatively compare thoracoscopic intrathoracic visualization between non-insufflated and 3 mmHg carbon dioxide (CO₂) insufflation in small dogs; (2) assess whether a single-incision subxiphoid approach under CO₂ insufflation provides sufficient access for complete and efficient inspection of all lung lobes, including the centrally located accessory lung lobe; and (3) assess the safety and feasibility of performing accessory lung lobectomy using a pretied loop ligature under 3 mmHg CO₂ insufflation. We hypothesized that low-pressure CO₂ insufflation would significantly enhance visualization compared to non-insufflation group, that single-incision subxiphoid access would permit complete lung exploration, and that accessory lung lobectomy using a pretied ligature loop would be feasible and safe for small dogs.

## 2 Materials and methods

### 2.1 Cadaver preparation

Eight canine cadavers, euthanized for reasons unrelated to this study, were used. Furthermore, the sample size (*n* = 8) was considered adequate to ensure experimental reproducibility and allow comparative analysis between procedures, consistent with standard practices in similar anatomical and surgical feasibility studies (14, 15, 17, 18). All cadavers were obtained with informed owner consent for post-mortem use in research. Review of medical records, together with gross inspection of the thoracic cavity prior to experimentation, revealed no evidence of pulmonary or other thoracic abnormalities. The Institutional Animal Care and Use Committee (IACUC; JBNU-NON2025-018) approved the use of these specimens. Cadavers were stored at −20 °C within 2 h of euthanasia and then thawed at 5 °C for 48 h before experimentation. To minimize tissue degradation, all procedures were completed within 3 weeks of initial storage.

### 2.2 Headings

**Figure 1:**
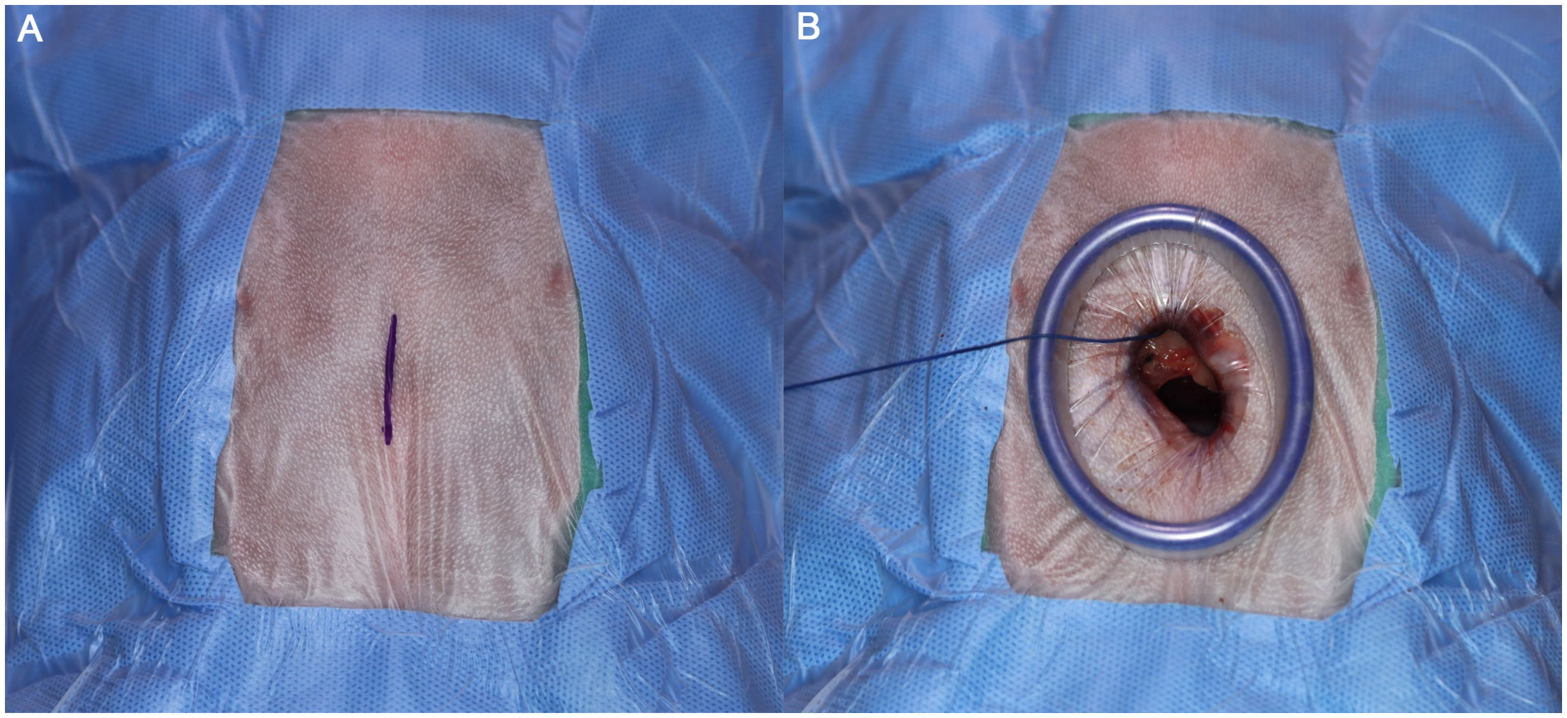
**(A)** Image of draped surgical site. Marked line indicates the incision line parallel to xiphoid process. **(B)** After the tunneling, wound retractor has been placed. For Visualization procedure, port lid was placed afterward. The Lapsingle consists of a wound retractor and a detachable multiport lid, which includes one 12-mm and three 5-mm ports.

Each cadaver was positioned in the ventrodorsal (VD) recumbency on a manually adjustable tilting surgical table (CUBI small, Tabtech s.r.o., Czech Republic), with the surgical field shaved from the first rib to the umbilicus. The forelimbs and hindlimbs were secured to the table, and the surgical field was draped to the center of the xiphoid process (Figure 1A). A 2.5-cm midline skin incision was made over the xiphoid process. Following blunt dissection of subcutaneous tissue, the xiphoid process was identified and used as a landmark. Dissection continued toward the pleural cavity using fingers or Metzenbaum scissors. Upon entering the pleural space, a single-port wound retractor (Lapsingle; Sejong Medical, Seoul, Korea) was inserted (Figure 1B). The Lapsingle consists of a wound retractor and a detachable multiport lid, which together form a single-port access system. The wound retractor provides a 64-mm wound diameter, and the lid includes one 12-mm and three 5-mm ports compatible with both 10-mm and 5-mm endoscopes.

### 2.3 Thoracic cavity visualization

**Figure 2:**
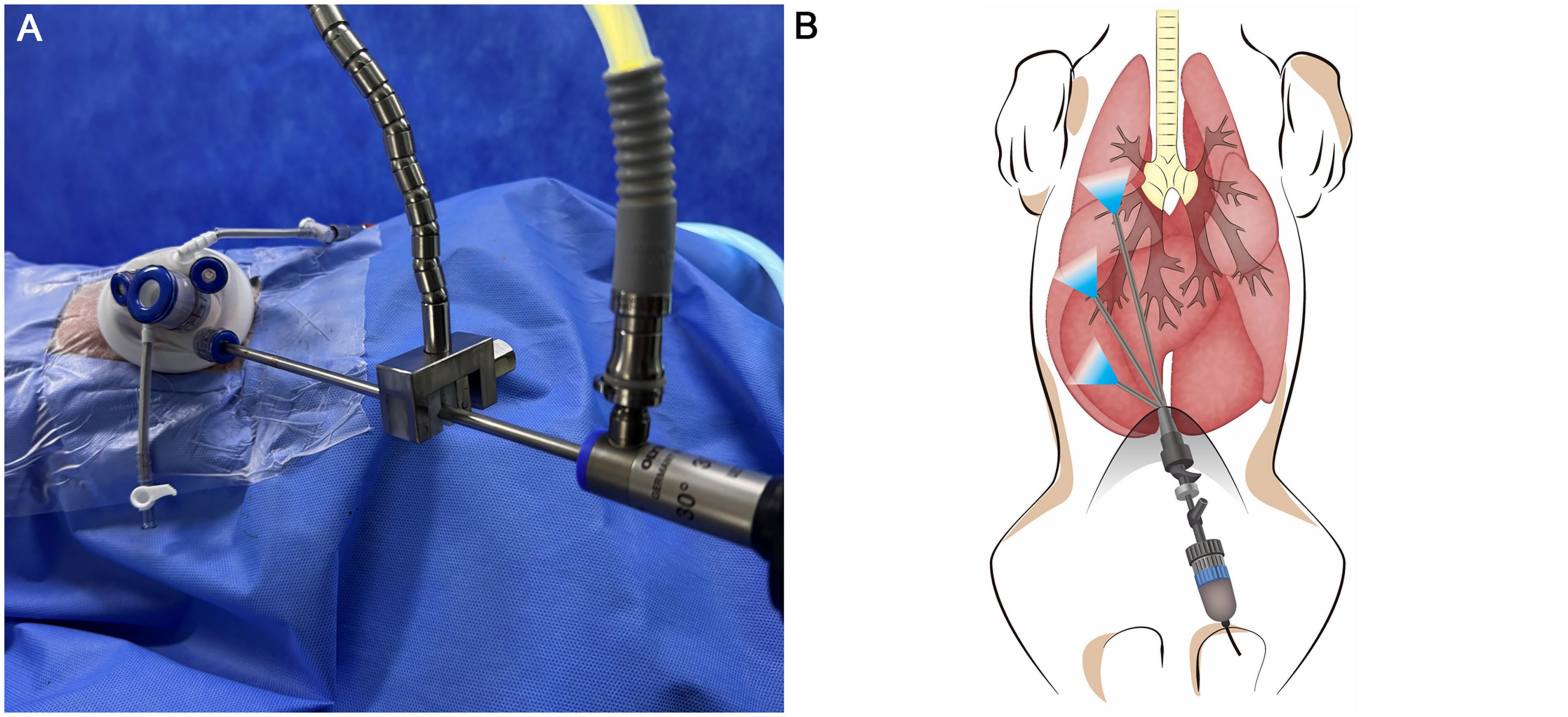
**(A)** Image of installed flexible arm during visualization procedure. **(B)** Illustration of right pleural visualization. Each pleural space was divided into three parts, Cranial, Middle, Caudal.

The table was tilted of 30° toward one and then the other side to facilitate visualization of the contralateral target hemithorax. The same angle was maintained for both right and left evaluations and was identical between the non-insufflation and 3 mmHg CO₂ insufflation conditions. The lid of the single-port access device was secured, and mechanical ventilation was initiated with a peak inspiratory pressure (PIP) of 15 cmH₂O, respiratory rate (RR) of 20 breaths per minute, and a positive end-expiratory pressure (PEEP) of 3 cmH₂O. A 5-mm laparoscopic scope (WA4KL530; Olympus, Tokyo, Japan) was introduced through a single port. Using retro-illumination as a guide, a needle was inserted into the thoracic cavity at the midpoint of the second intercostal space to visualize the cranial portion of the right pleural cavity. Care was taken to avoid lung contact and prevent iatrogenic pneumothorax. Once the needle was correctly positioned, the camera angle was adjusted to center the needle within the field of view. The laparoscope was stabilized using a flexible arm (Figure 2A). The camera was then retracted posteriorly until the lung edge appeared at the lower margin of the visual field. After confirming optimal positioning, video recording was initiated. At least three complete respiratory cycles were captured before recording was paused. Images were saved as the non-insufflation group (NIG). Subsequently, the pleural cavity was insufflated to 3 mmHg CO₂, and the recording procedure was repeated. These images were saved as the 3 mmHg CO₂ insufflation group (3CIG). This method for quantitative visualization assessment was adapted from the approach described by previous study (12). The process was repeated at the second, fifth, and seventh intercostal spaces, corresponding to the cranial, middle, and caudal pleural segments, respectively, to visualize the entire right pleural cavity (Figure 2B). After completing the right-side assessment, the table was tilted to assess the cranial, middle, and caudal aspects of the left hemithorax. This procedure was repeated to assess the cranial, middle, and caudal aspects of the left pleural cavity.

**Figure 3:**
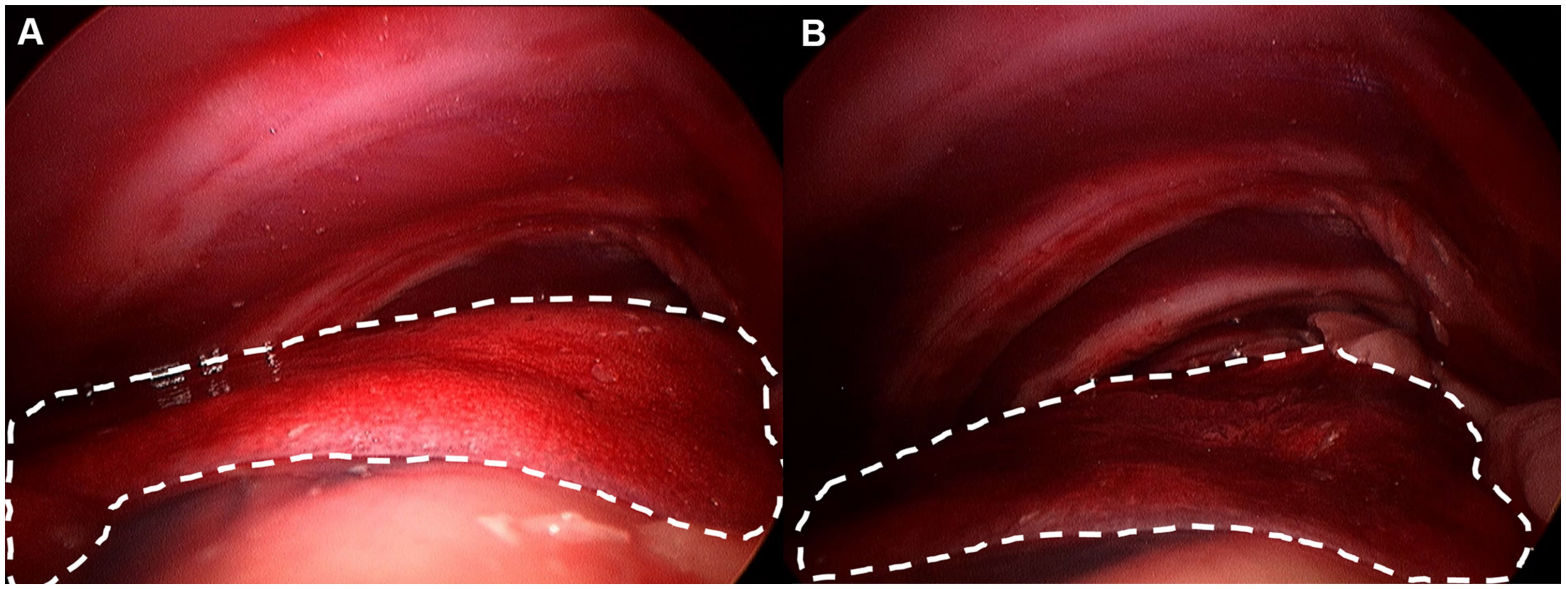
Lung margin was drawn by imaging software. Each lung margin was drawn at least three times and average was considered lung area. **(A)** Non-insufflation group. **(B)** 3 mmHg CO2 insufflation group.

Captured images were processed using ImageJ software (version 1.49; National Institutes of Health, Bethesda, Maryland, United States) to quantitatively evaluate thoracic cavity visualization. For each frame, the total endoscopic field area was delineated, and the lung margins were manually traced along the visible lung lobes (Figure 3). This procedure was repeated three times, and the average was designated as the zone of interest. The traced lung area was divided by the total frame area to calculate the visual obstruction ratio, also referred to as the lung collapsing ratio, representing the percentage of the endoscopic view occupied by lung tissue. As in previous studies, the intrathoracic working space was quantitatively evaluated using this ratio, with lower obstruction values indicating a larger available working space (12). This parameter was used to compare visualization between NIG and 3CIG.

### 2.4 Exploration

**Figure 4:**
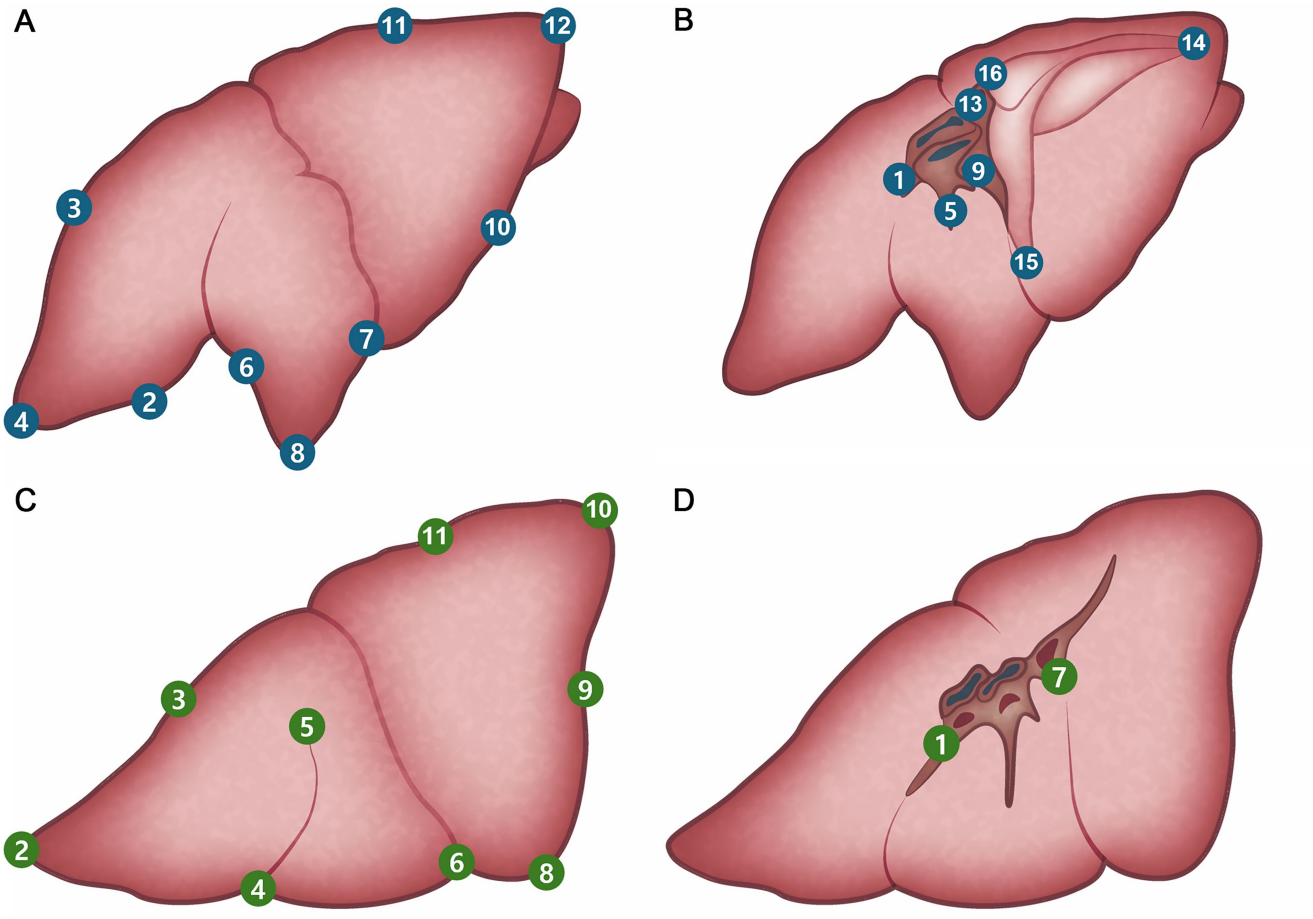
Pre-planned marking site of lung. **(A)** Right lateral side. **(B)** Right medial side. **(C)** Left lateral side. **(D)** Left medial side.

**Figure 5:**
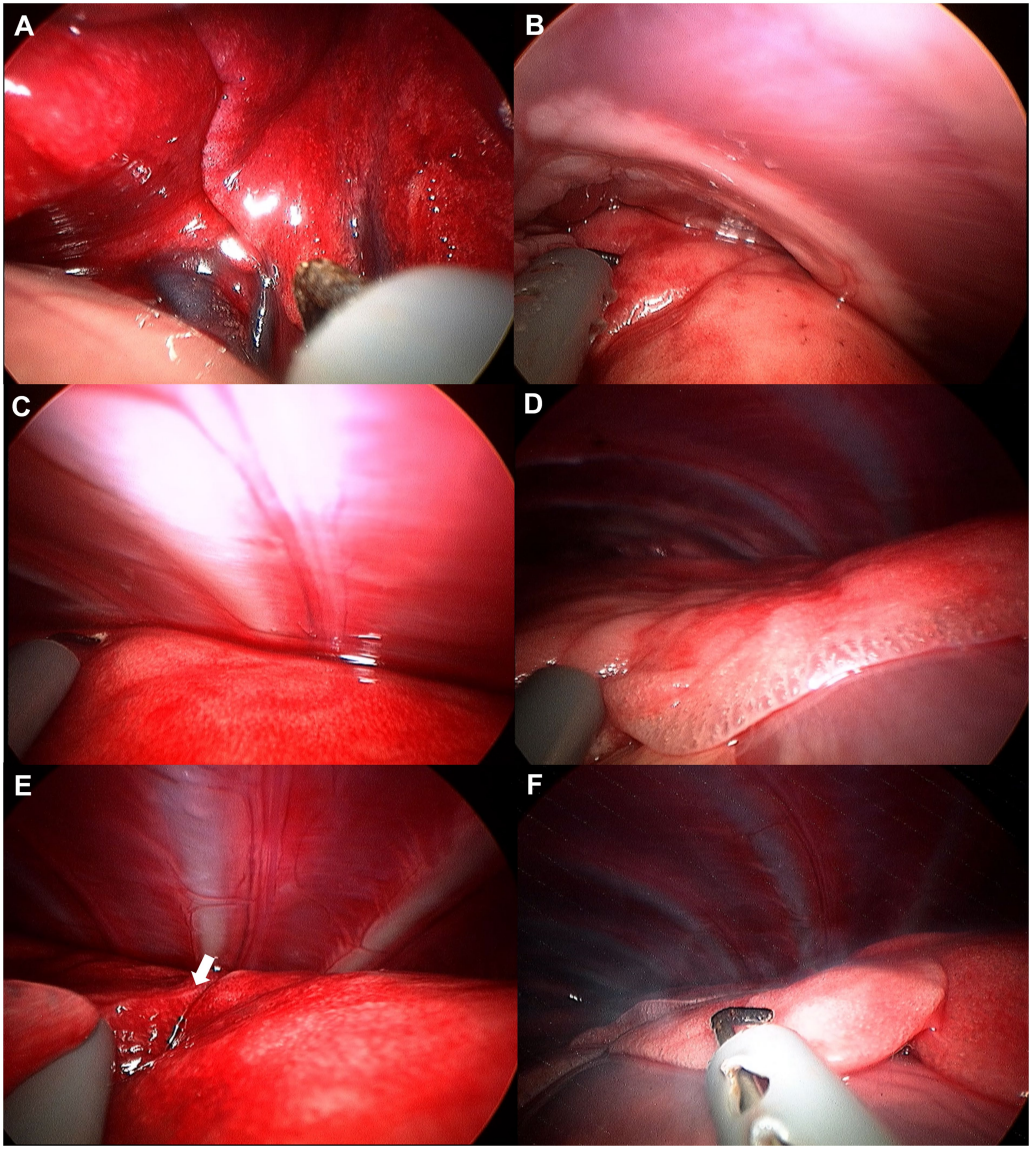
Image of left cranial lobe exploration procedure. **(A)** Hilus. **(B)** Cranial apex of cranial part. **(C)** Dorsal edge. **(D)** Cranial apex of caudal part. **(E)** Bifurcation (white arrow). **(F)** Caudal apex of caudal part.

**Figure 6:**
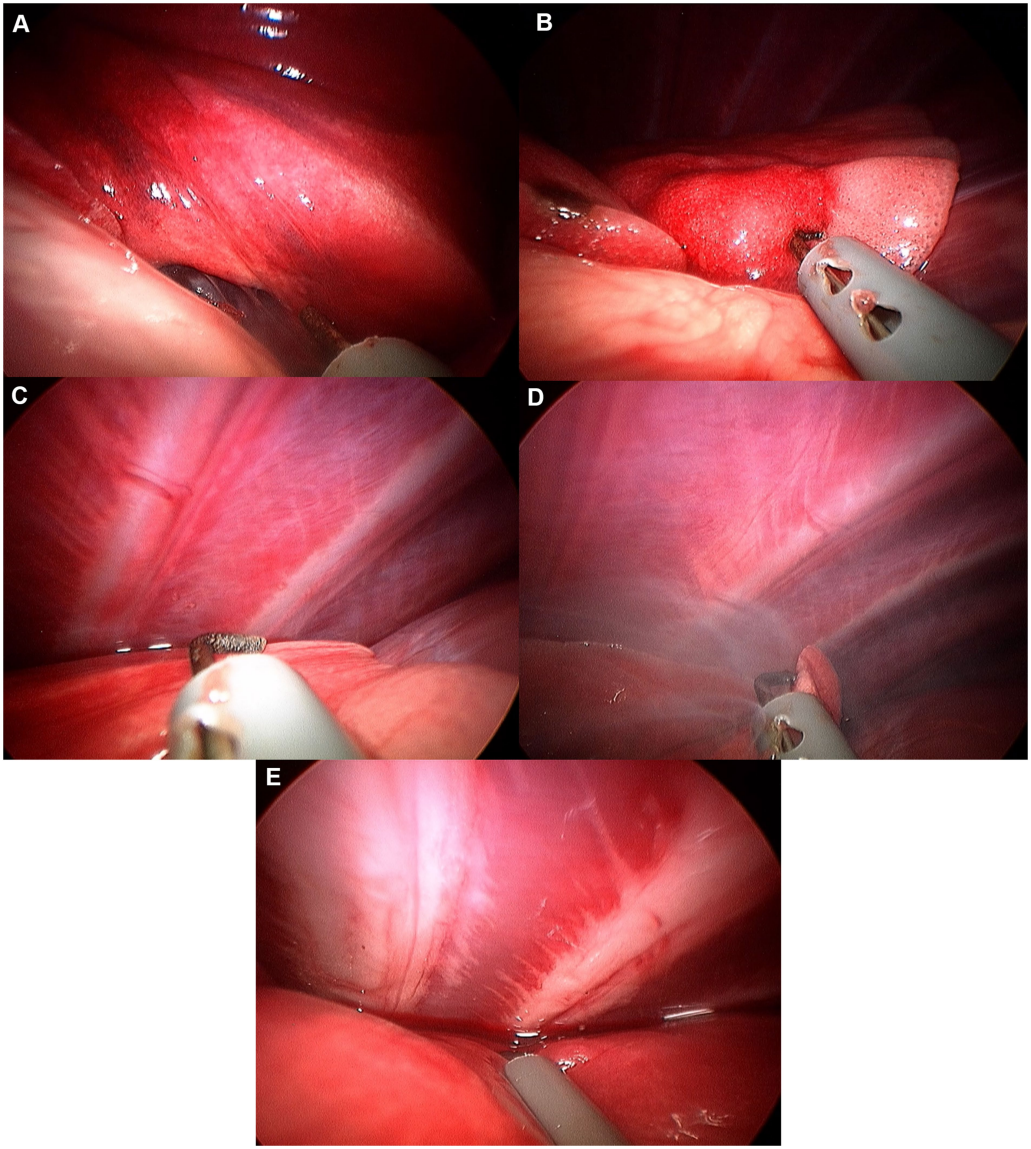
Image of left caudal lobe exploration procedure. **(A)** Hilus. **(B)** Ventral apex. **(C)** Caudal edge. **(D)** Caudal apex. **(E)** Dorsal edge.

**Figure 7:**
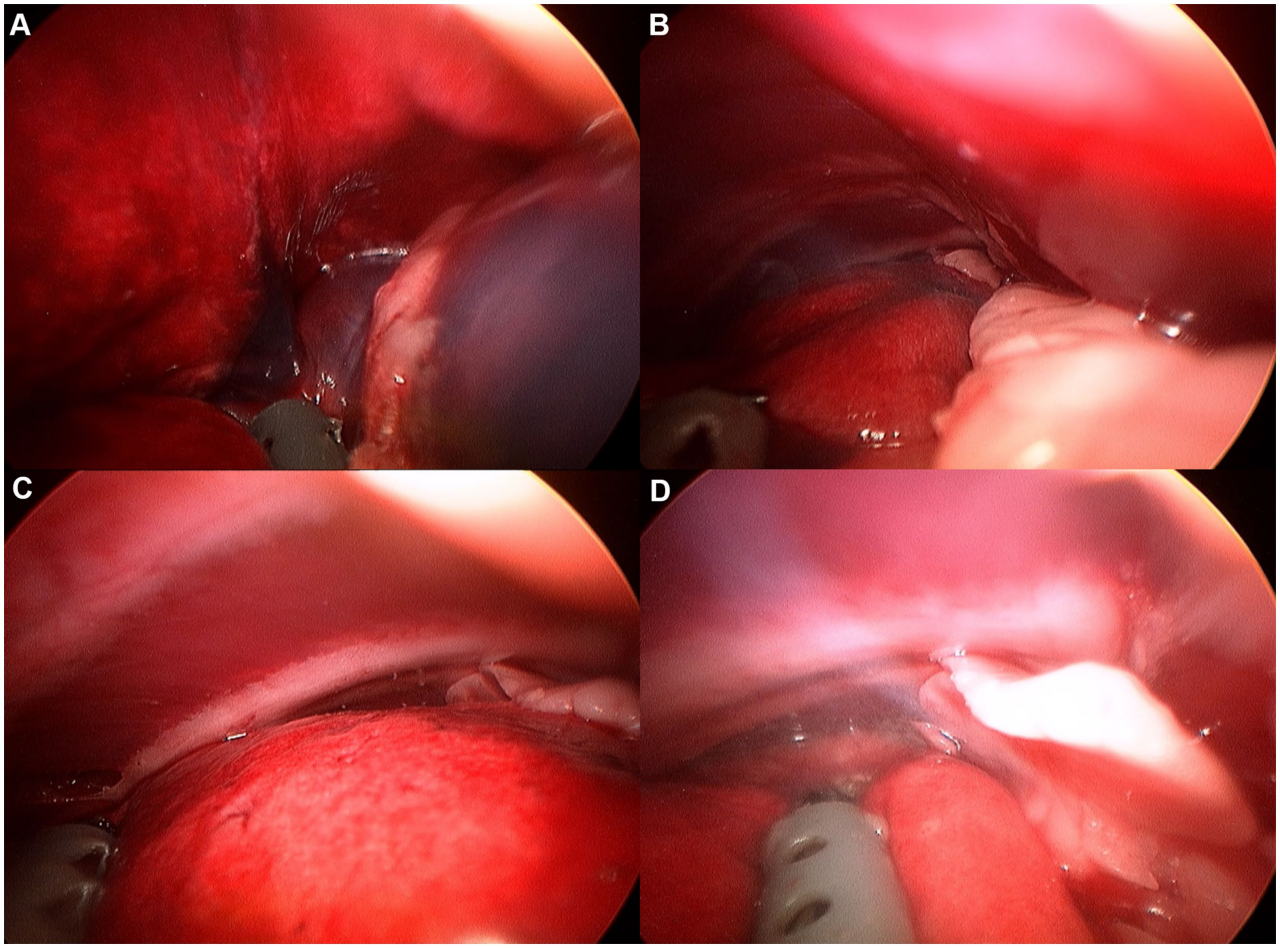
Image of right cranial lobe exploration procedure. **(A)** Hilus. **(B)** Ventral edge. **(C)** Dorsal edge. **(D)** Cranial apex.

**Figure 8:**
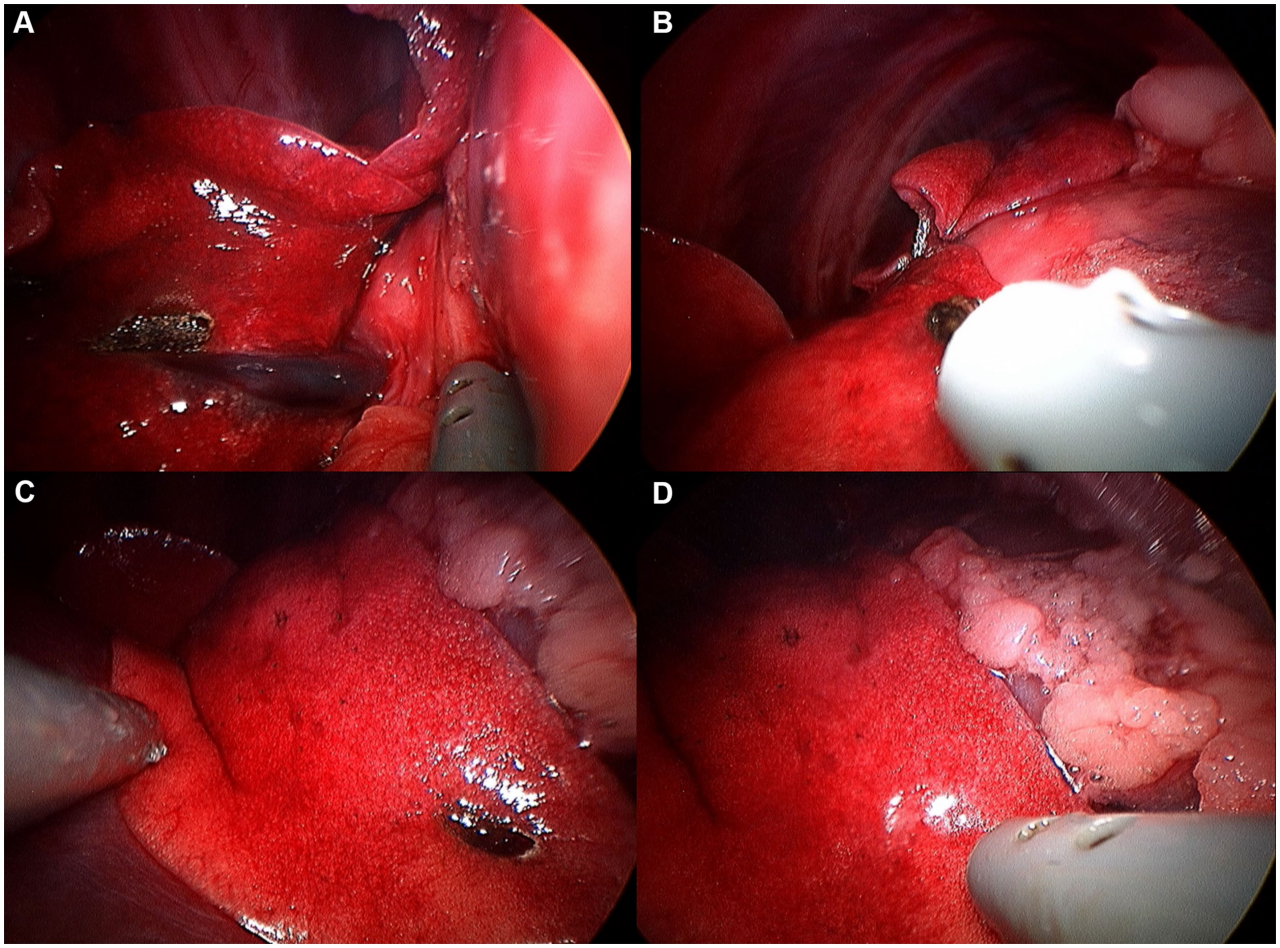
Image of right middle lobe exploration procedure. **(A)** Hilus. **(B)** Cranial edge. **(C)** Caudal edge. **(D)** Ventral apex.

**Figure 9:**
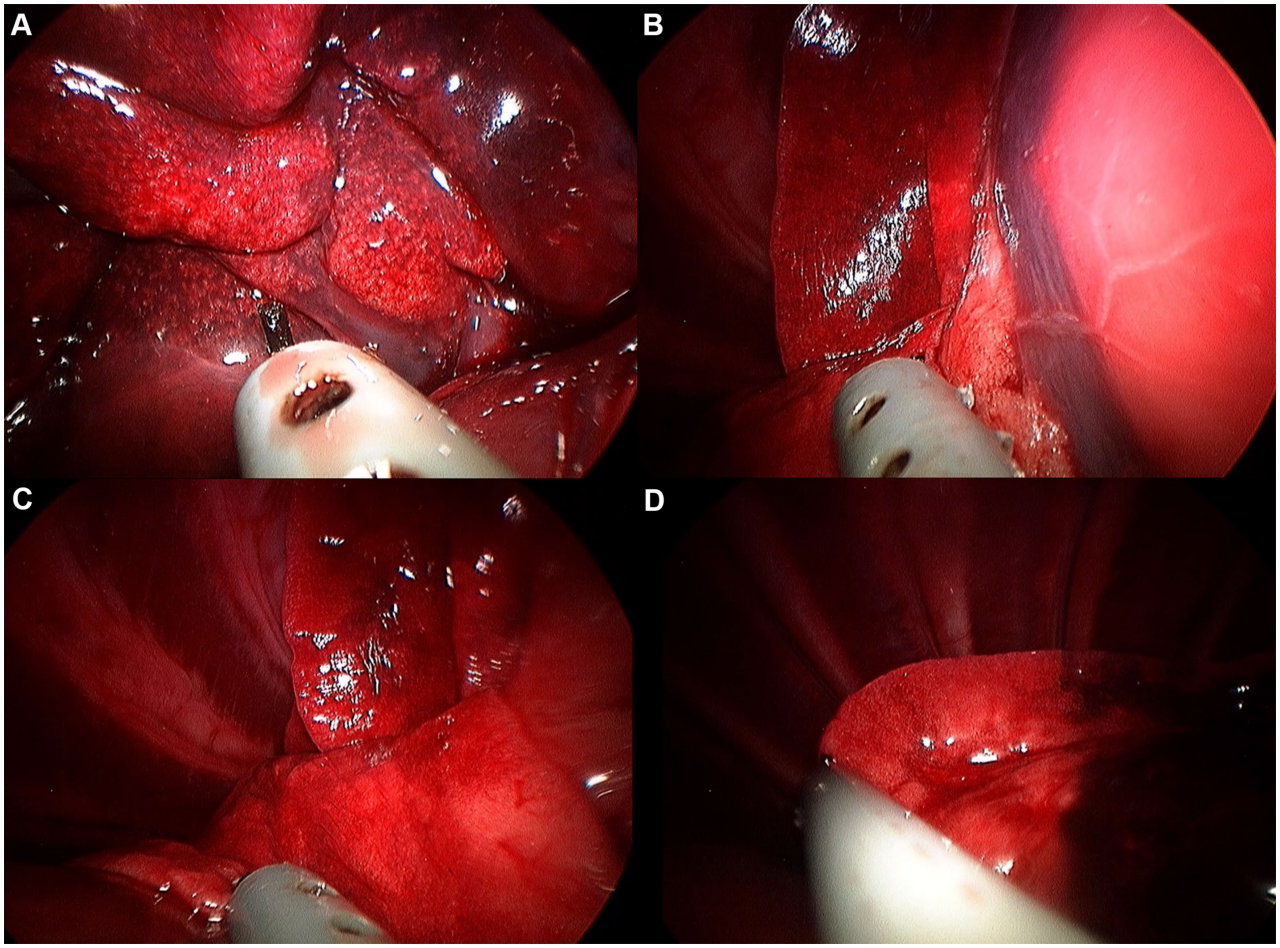
Image of right caudal lobe exploration procedure. **(A)** Hilus. **(B)** Ventral edge. **(C)** Dorsal edge. **(D)** Caudal apex.

**Figure 10:**
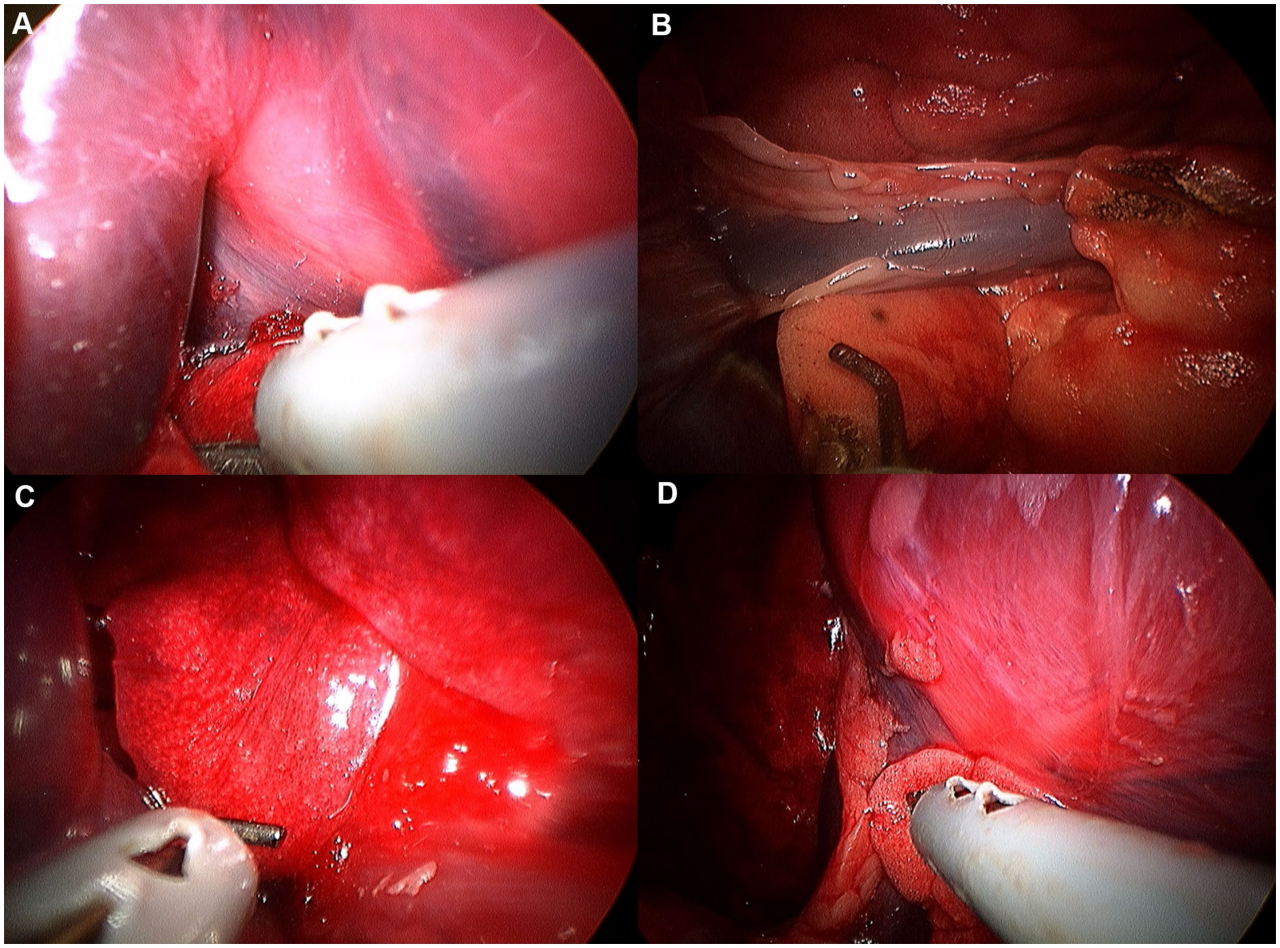
Image of accessory lobe exploration procedure. **(A)** Hilus. **(B)** Caudal apex. **(C)** Ventral apex. **(D)** Dorsal apex.

The exploration procedure was performed from the left to the right hemithorax. Starting with the left cranial lobe, each predesignated site (Figure 4; Tables 1, 2) was marked using a laparoscopic monopolar instrument in coagulation mode, which was used only for marking. The exploration and scoring were performed only under 3CIG to obtain the quantitative results of visualization achieved with CO₂ insufflation. The cranial and caudal portions of the cranial lobe, followed by the caudal lobe, were marked sequentially (Figures 5, 6). After completing the left side, the right hemithorax, including the cranial, middle, caudal, and accessory lung lobes, was explored similarly (Figures 7–10). The time required to explore each lobe was recorded. Each marking point was allotted 1 min; therefore, a lobe with four planned marking sites was allocated 4 min each. If the task was completed within the allotted time, the lobe received a full score of four points. If the total time exceeded the allocation, one point was deducted for every additional 2 min.

**Table 1:** Pre-planned landmarks of right lung lobe exploration.

Right lobes		
Cranial lung lobe	1	Hilus
	2	Ventral edge
	3	Dorsal e dge
	4	Cranial apex
Middle lung lobe	5	Hilus
	6	Cranial edge
	7	Caudal edge
	8	Ventral apex
Caudal lung lobe	9	Hilus
	10	Ventral edge
	11	Dorsal edge
	12	Caudal apex
Accessory lung lobe	13	Hilus
	14	Caudal apex
	15	Ventral apex
	16	Dorsal apex

**Table 2:** Pre-planned landmarks of left lung lobe exploration.

Left lobes		
Cranial lung lobe	1	Hilus
	2	Cranial apex of cranial part
	3	Dorsal edge
	4	Cranial apex of caudal part
	5	Bifurcation
	6	Caudal apex of caudal part
Caudal lung lobe	7	Hilus
	8	Ventral apex
	9	Caudal edge
	10	Caudal apex
	11	Dorsal edge

### 2.5 Accessory lung lobectomy

**Figure 11:**
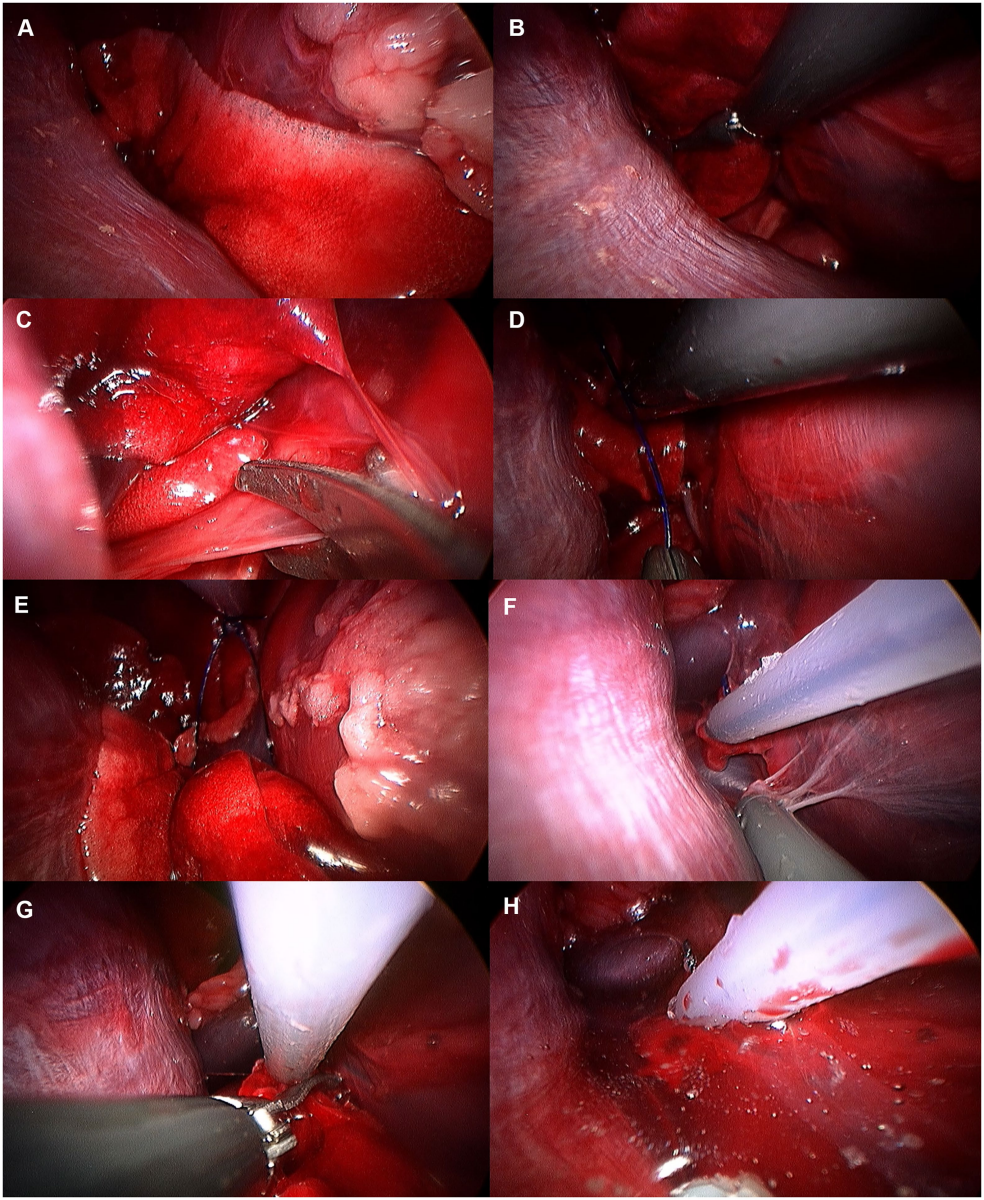
Image of accessory lung lobectomy using pretied ligature. **(A)** Accessory lung lobe before procedure. **(B)** Lung lobe was handled with non-crushing forceps (e.g., babcock forceps). **(C)** Pulmonary ligament was cut using metzenbaum scissors or monopolar device. **(D)** Pretied ligature was handled using maryland forceps or right angle forceps. **(E)** Lung lobe was passed through the ligature loop. **(F)** After making sure lung lobe was totally passed through the ligature loop, ligature was tied. **(G)** Tied lung lobe was cut 1–2 mm distal to the ligation. **(H)** Leak test was performed before closure.

Accessory lung lobectomy was performed following exploration under 3CIG (Figure 11). The lungs were gently retracted using non-crushing forceps to avoid parenchymal injury. To access the accessory lung lobe, the mediastinal recess was carefully opened using Metzenbaum scissors and blunt dissection with endoscopic forceps. The pulmonary ligament attached to the accessory lung lobe of the mediastinum was dissected using Metzenbaum scissors or a laparoscopic monopolar instrument to preserve the surrounding structures. Once the accessory lung lobe was mobilized, a 2–0 polydioxanone pre-tied ligature loop (PLL) (Endoloop PDS II, ETHICON, United States) was introduced through the port. Maryland forceps were used to grasp and position the loop around the base of the accessory lobe. After confirming proper positioning in the hilar region, the loop was tightened, and the lobe was transected 5-10 mm distal to the ligature using Metzenbaum scissors (17, 19). To assess air leakage, the thoracic cavity was filled with sterile normal saline, and the lungs were ventilated through the circuit for 30 s at a peak inspiratory pressure of 20 cmH₂O. The resection site was observed endoscopically, and the procedure was completed after confirming the absence of air leakage.

### 2.6 Necropsy

A necropsy was performed upon completion of the procedures. Lung lobes were separated from the trachea, and marked areas were examined and scored. An area was scored as 1 if marked in the correct position and 0 otherwise. The lateral side of the dorsal margin was scored as 1 if marked at more than 2/3 of the lobe’s height. The exploration score (ES) was calculated by summing the marked score and the time score per lobe, then dividing by twice the marking score to compare lobes. Values approaching 1 were considered indicative of easier exploration, whereas values approaching 0 indicated greater difficulty. After ES assessment, an endotracheal tube was placed in the trachea and sealed using cable ties. The tube was then fitted with a sphygmomanometer, and the lobes were submerged in water to quantitatively assessed leakage at physiological lung pressure (15 mmHg).


$$\text{Exploration score}=\frac{\text{Time}+\text{Marked point}}{2\times \text{Marking point}}$$

### 2.7 Statistical analysis

Experimental results were organized using Microsoft Excel. Statistical analysis was performed using GraphPad Prism (GraphPad Software, UK). The Shapiro–Wilk test assessed normality, and the paired *t*-test compared NIG and 3CIG. *p*-values < 0.05 were considered statistically significant.

## 3 Results

### 3.1 Animals

Eight canine cadavers were evaluated, with beagles being the most common breed (*n* = 4), followed by mixed breeds (*n* = 2), Cocker Spaniels (*n* = 1), and Pomeranians (*n* = 1). Intact male dogs were the most represented (*n* = 4), followed by spayed female dogs (3) and castrated male dogs (*n* = 2). The mean body weight was 7.89 kg (range: 6–10 kg), and the mean age was 32 months (range: 21–58 months).

### 3.2 Thoracic cavity visualization

Quantitative evaluation of intrathoracic visualization revealed that 3CIG demonstrated significantly increased intrathoracic working space and improved visibility in all six thoracic segments compared to NIG, as evidenced by a lower lung obstruction ratio (Table 3). In the right cranial segment, visual obstruction ratio was significantly lower in 3CIG (0.3094; range: 0.2243–0.4053) than in NIG (0.4109; range: 0.2485–0.5334; *p* = 0.0008). In the right middle segment, the 3CIG mean was 0.2106 (range: 0.1241–0.3173), significantly lower than the NIG mean of 0.2891 (range: 0.1784–0.4211; *p* < 0.0001). In the right caudal segment, the 3CIG mean was 0.1253 (range: 0.0833–0.2027), compared with 0.2156 for NIG (range: 0.1153–0.2666; *p* < 0.0001). For the left cranial segment, the 3CIG mean was 0.2277 (range: 0.1808–0.2764), significantly lower than the NIG mean of 0.3151 (range: 0.2137–0.4473; *p* = 0.0021). In the left middle segment, the 3CIG mean was 0.1280 (range: 0.0410–0.2017), compared with 0.2424 for NIG (range: 0.0980–0.3699; *p* = 0.0002). Finally, in the left caudal segment, the 3CIG mean was 0.1550 (range: 0.0747–0.2361), significantly lower than the NIG mean of 0.2222 (range: 0.1169–0.2728; *p* = 0.0002).

**Table 3:** Average of visualization score of each area.

	Right		Left	
Area	NIG	3CIG	NIG	3CIG
Cranial area	0.4109	0.3094	0.3151	0.2277
Middle area	0.2891	0.2106	0.2424	0.1280
Caudal area	0.2156	0.1253	0.2222	0.1550

In every group, 3 mmHg CO_2_ insufflation group (3CIG) showed significantly low visualization score than non-insufflation group (NIG).

*n* = 8, *p* < 0.05.

### 3.3 Exploration

Mean exploration times and corresponding ES for each lung lobe under 3CIG are presented in Table 4. In the right cranial lobe, the mean exploration time was 218 s (range: 144–323 s), with a mean ES of 0.94. The right middle lobe was explored at 207 s (range: 157–257 s), with a mean ES of 0.98. The right caudal lobe required a mean of 421 s (range: 238–824 s) and had a mean ES of 0.73. The accessory lung lobe required the longest exploration time, with a mean of 587 s (range: 443–737), and had the lowest mean ES of 0.59. On the left side, the left cranial lobe was explored in a mean of 408 s (range: 273–534 s), with a mean ES of 0.91. The left caudal lobe required a mean of 439 s (range: 187–594 s) and had a mean ES of 0.81 (Table 5).

**Table 4:** Average of exploration time of each lung lobe.

Parameter	Right lobe		Left lobe	
Exploration time (second)	Cranial	218	Cranial	408
	Middle	207		
	Caudal	421	Caudal	439
	Accessory	587		

**Table 5:** Average of exploration score of each lung lobe.

Parameter	Right lobe		Left lobe	
Exploration score	Cranial	0.94	Cranial	0.91
	Middle	0.98		
	Caudal	0.73	Caudal	0.81
	Accessory	0.59		

Exploration score is ranged from 0 to 1, which values close to 1 is regarded as easier exploration.

### 3.4 Accessory lung lobectomy

Accessory lung lobectomy was performed with a mean duration of 921 s (range: 582–1,175 s; *n* = 8). Hilar resection was successfully completed in all cases. No air leakage was detected during the leakage test conducted before finalizing the procedure or during necropsy (Table 6).

**Table 6:** Result of accessory lung lobectomy using pretied ligature.

Variables	Accessory lung lobectomy (*n* = 8)
Mean duration (second)	921
Hilus resection (n)	8
Air leakage (n)	0

In every case, lobectomy was successfully performed without air leakage.

## 4 Discussion

Intrathoracic visualization was achieved using a single-port thoracoscope inserted via the subxiphoid approach. Comparison of the NIG and the 3CIG revealed significantly improved visibility with low-pressure CO₂ insufflation across all pleural zones. The low-pressure CO₂ setting improved visibility in every pleural zone examined. Lung tissue obscuration was reduced by approximately 24.7% in the right cranial region, 27.2% in the right middle region, and 41.9% in the right caudal region, with the latter showing the most pronounced improvement on the right side. On the left side, the corresponding cranial, middle, and caudal regions show gains of 27.7, 47.2, and 30.2%, respectively, with the left middle region exhibiting the greatest overall enhancement. These findings confirm that low-pressure (3 mm Hg) capnothorax reliably expands the working space throughout the thorax, particularly in the left middle and right caudal lobes, which may facilitate instrument maneuverability. To the best of our knowledge, no previous canine study has provided objective numerical evidence of such visual improvement at this pressure level. Given the documented challenges of one-lung ventilation in toy-breed dogs (10, 11), low-pressure CO₂ insufflation is supported as a practical alternative for clinical use in this population. Previous studies have reported that the subxiphoid port in the VD recumbency provides easy access to the mediastinum and broad visualization of the thoracic cavity (4, 13, 14). In one study, single-incision thoracoscopic lung lobectomy was performed in beagle dogs, with improved visualization of midline and central lung regions compared with the traditional lateral approach (13). Furthermore, the lateral thoracoscopic approach, commonly used for lung lobectomy, presents challenges in visualizing centrally located structures, such as the accessory lung lobe (9). In this study, all lung lobes were successfully explored without additional ports using the subxiphoid single-incision approach. These findings align with prior literature and suggest that the subxiphoid approach is a viable alternative, particularly for procedures involving the accessory lung lobe. However, ES varied significantly among lobes. For example, the right cranial lobe had an average ES of 0.94, the right middle lobe 0.98, the right caudal lobe 0.73, the accessory lung lobe 0.59, the left cranial lobe 0.91, and the left caudal lobe 0.81. These differences likely result from the anatomical location of each lung lobe and the presence of adjacent structures, such as the heart, great vessels, and diaphragm, which can impede instrument manipulation. The right caudal, accessory, and left caudal lobes, located more caudally and closer to the port site, required significantly longer exploration times than cranial lobes. Previous studies attribute such delays to limited instrument trajectory adjustment and the occurrence of dead angles when visualizing structures located near the port (20–22). Proximity to the port constricts the viewing angle and increases the risk of instrument-camera collisions hindering accurate inspection. In our study, visualization failed twice for the lateral dorsal margins of the right and left cranial lobes, which, despite being farther from the port, lie in the dorsolateral sector where scope angle adjustment is challenging. Previous reports suggest that variable-angle scopes or articulating instruments can minimize dead angles and improve maneuverability in complex anatomical zones (15). Future studies should evaluate whether these devices can address the visual limitations observed in single-incision subxiphoid thoracoscopy in small-breed dogs. The accessory lung lobe, located adjacent to the diaphragm, heart, and caudal vena cava (CVC), is notoriously difficult to visualize using the conventional lateral thoracoscopic approach (8). This limitation was overcome by employing the subxiphoid single-incision approach, enabling successful lung lobectomy in all eight dog cadavers. Surgical staplers, commonly used in lung lobectomy, are relatively large and challenging to manipulate or angle appropriately within the limited thoracic cavity of small dogs. Smaller instruments are preferred in conditions (14). In contrast, the PLL is compact, easier to handle in confined spaces, and more cost-effective than staplers. Accessory lung lobectomy was performed using the PLL, and no air leakage was detected during leakage testing. This indicates that the PLL, combined with the subxiphoid single-incision approach, enables effective vascular and bronchial ligation, even in the technically challenging accessory lung lobe. Previous studies have noted poor visualization of the accessory lung lobe with lateral approaches (9). In contrast, in our study, the subxiphoid approach facilitated visualization of caudal thoracic structures, including the accessory lung lobe, and enabled effective lobectomy in all cadavers, despite the limited working space in small-breed dogs. The PLL has a noticeably slimmer profile than surgical staplers and is easier to handle, reducing instrument crowding and mitigating the restricted angulation often encountered in tight thoracic spaces—observations consistent with earlier reports. Previous studies have also shown that bronchial closure with these loops prevents postoperative air leakage during thoracoscopic lobectomy (17, 18). Our findings support and extend this evidence by confirming that this method yields a secure, dependable lobectomy in practice. Because loops are inexpensive and easy to deploy, they represent a practical alternative in clinics where stapling devices are unavailable or cost-prohibitive. Although larger equipment—such as linear staplers or multiple ligatures—may still be necessary for transecting sizable vessels or bronchi in medium- and large-breed dogs, our results indicate that PLL is safe and effective option for limited resections, including accessory lung lobe removal, in small-breed patients. This study has several limitations. The use of cadaveric models precluded evaluation of physiological responses, such as hemodynamic changes, respiration, and postoperative recovery. In particular, the absence of a beating heart did not allow assessment of potential cardiac displacement or hemodynamic effects, such as arrhythmia or hypotension, that may occur during manipulation or ligation of the accessory lung lobe in live animals. Although prior studies indicate that low-pressure CO₂ insufflation (3 mmHg) provides improved visualization without significant cardiovascular side effects, physiological responses during accessory lung lobectomy, particularly near critical structures like the diaphragm and CVC, may differ in live animals. Furthermore, vascular sealing performance could not be evaluated in cadaveric models. All procedures were performed on macroscopically healthy lungs, which may not fully represent surgical conditions involving pathological or friable pulmonary tissue. Additionally, only eight cadavers of small-breed dogs were included, limiting the generalizability of the results to other breeds, weights, or pleural cavity configurations. Finally, as a single surgeon performed the procedures, variability in surgical skills or learning curve progression could not be assessed.

## 5 Conclusion

In this study, we demonstrated that 3 mmHg carbon dioxide insufflation significantly improved thoracoscopic visibility compared to non-insufflation in small-breed dogs. Using a subxiphoid single-incision approach, complete exploration of all lung lobes was achieved, and accessory lung lobectomy was safely and effectively performed using a pretied ligature. These findings quantitatively validate the benefits of low-pressure CO₂ insufflation and support the feasibility of subxiphoid single-port thoracoscopy as a promising minimally invasive technique for thoracic surgery in small dogs.

## Data Availability

The original contributions presented in the study are included in the article/supplementary material, further inquiries can be directed to the corresponding authors.
